# Heart Rate Variability Frequency Domain Alterations among Healthy Nurses Exposed to Prolonged Work Stress

**DOI:** 10.3390/ijerph15010113

**Published:** 2018-01-11

**Authors:** Rossana Borchini, Giovanni Veronesi, Matteo Bonzini, Francesco Gianfagna, Oriana Dashi, Marco Mario Ferrario

**Affiliations:** 1 Research Centre in Epidemiology and Preventive Medicine (EPIMED), Department of Medicine and Surgery, University of Insubria, Via O Rossi 9-Pad Rossi-1 Piano, 21100 Varese, Italy; rossana.borchini@asst-settelaghi.it (R.B.); giovanni.veronesi@uninsubria.it (G.V.); francesco.gianfagna@uninsubria.it (F.G.); 2 Medicina del Lavoro, Preventiva e Tossicologia, ASST dei Sette laghi di Varese, 21100 Varese, Italy; 3 Department of Clinical Sciences and Community Health, Università degli Studi di Milano, 20122 Milan, Italy; matteo.bonzini@unimi.it; 4 Department of Epidemiology and Prevention, IRCCS Istituto Neurologico Mediterraneo NEUROMED, 86077 Pozzilli, Italy; 5 School of Specialization in Occupational Medicine, University of Insubria, 21100 Varese, Italy; oriana.dashi@gmail.com

**Keywords:** work stress, heart rate variability, frequency-domain, ECG-monitoring, nurses, longitudinal study, parasympathetic nervous system

## Abstract

The deregulation of the autonomic nervous system assessed through the heart rate variability (HRV) analysis is a promising pathway linking work stress and cardiovascular diseases. We aim to investigate the associations between HRV High Frequency (HF) and Low Frequency (LF) powers and work stress in a sample of 36 healthy nurses. Perceived work stress was assessed twice one year apart, using the Job Content and Effort Reward Imbalance questionnaires. This allows to classify nurses in three exposure groups: “prolonged high stress” (PHS), “recent high stress” (RHS) and “stable low stress” (SLS). A 24-h ECG monitoring was later performed during a working day (WD) and a subsequent resting day (RD). Statistically significantly lower (*p* < 0.02) HF and LF means were found in PHS and RHS nurses during the working periods. In the subsequent resting periods, HF means showed increases over time in the RHS (beta = +0.41, *p* < 0.05), but not in PHS nurses. LF means did not show any substantial increases in the resting periods, in the PHS group with geometric means lower when compared to SLS, in the non-working and resting periods. Our study evidences that both prolonged and recent perceived high work stress were associated with a reduction of HF and LF powers during work. In addition, prolonged stress was associated with a lack of recovery during not-working and resting periods.

## 1. Introduction

Psychological work stress has been associated with the development of coronary heart disease [1,2,3]. Two major pathways have been advocated to explain the effects of psychological stress on the cardiovascular system: the hypothalamic-pituitary-adrenal axis and the autonomic nervous system [4,5]. Heart rate variability (HRV), which represents the variations of normal RR intervals in the time and in the frequency domains, is a non-invasive tool used to assess the autonomic nervous system control on the heart rate [6]. In particular, in the time domain, the standard deviation of normal RR intervals (SDNN), the root mean square successive differences (RMSSD) and the percentage of adjacent cycles that are greater than 50 ms (pNN50) are considered as indices of vagal activity. In the frequency domain, the high frequency (HF: 0.15–0.40 Hz) power characterizes the parasympathetic control and the low frequency (LF: 0.04–0.15 Hz) both the sympathetic and parasympathetic activities [6].

HRV variations characterise the early effects on the heart of the imbalance between the sympathetic and the vagal systems [7]. HRV modifications as decrease of indexes of vagal tone in time and frequency domains showed independent associations with increased mortality and morbidity [8,9]. Years ago, Kleiger et al. showed that the reduced HRV was a significant and independent predictor of mortality in post-myocardial infarction patients [10]. Since then, consistent evidence supports the notion that a decrease in the vagal activity, as indexed by HRV alterations, predicts mortality in high CVD risk as well as low CVD risk populations [11,12,13,14,15].

Perceived work stress has repeatedly been shown to be associated with the incidence of coronary heart disease, when characterised either by the demand-control [1,16,17] or the Effort–Reward Imbalance (ERI) [18] models. These single-study results were confirmed by a meta-analysis in which subjects were classified at high work stress by both models [19]. Among high CVD risk workers including those exposed to elevated work stress, non-invasive ECG monitoring may be clinically relevant to early detect HRV alterations.

Several studies have investigated the association between work stress and the decrease of the time domain HRV parameters in apparently healthy employees [20,21,22,23,24,25,26,27]. Chandola et al. found a lower SDNN in subjects with a low job control, in a large sample of English civil servants [20,21]. Wrikotte et al., in a sample of 109 Dutch healthy white-collar males, recorded a lower RMSSD in workers with a high ERI imbalance [22]. Collins et al. found an inverse significant correlation between high strain and SDNN in a sample of 36 American healthcare male employees [23]. Clays et al. reported a correlation between high job strain and the pNN50 in a large sample of Belgian blue and white-collar males [24]. It is worth mentioning that Riese et al. [28] did not find any changes in RMSSD between different levels of job strain nurses, based on one year repeated exposure assessments, and Hintsanen et al. found RMSSD and pNN50 reductions in stressed women, but not in men [29]. We previously reported [27] lower SDNN and SDNN Index values, but not RMSSD and pNN50 in nurses characterized by a prolonged high job strain when compared to stable low strain subjects. 

The studies that have explored the association between work stress and the frequency domain (FD) HRV parameters report contradictory results [20,21,23,24,25,26,29,30,31,32]. The large majority of these findings come from a cross-sectional study design, which did not allow to explore the effects of prolonged high work stress conditions. The purpose of this research is to assess the modifications of HRV FD parameters in a sample of healthy nurses exposed to recent and prolonged perceived work stress in comparison to nurses in stable low work stress, during the work period (WP) in a working day (WD). Moreover, we investigate the recovery of FD HRV alterations during the non-working period (NW) in the same working day and in a following morning of a resting day (RD).

## 2. Materials and Methods

### 2.1. Study Sample

A sample of nurses and nurse assistants, employed full-time (36 h per week) since at least one year in a Northern Italian university hospital, were invited to participate in a survey on working conditions and work stress, as elsewhere further described [27]. The survey took place in February 2010, and 398 out of 518 nurses (participation rate 76%) filled in structured questionnaires for work stress assessment, described below. Twelve months apart, all nurses were asked to fill in the same structured questionnaires, and 313 participated. All recruited subjects signed an informed consent, and the survey was a part of the assessment of stress at work required by the Italian legislation [33].

### 2.2. Job Strain Assessment

The perceived work stress conditions were assessed using both the Job Content Questionnaire (JCQ) [17,27] and the Effort–Reward Imbalance questionnaire [18,27]. In each survey, subjects were categorized at “high stress” if the psychological job demand (PJD) score was above the second sample-specific tertile value (>38) and the decision latitude (DL) score was below the first tertile (<66); or if the ERI ratio (the ratio between Effort and Reward scores) was above the second tertile (>1.21). Similarly, “low stress” subjects were identified when either PJD was below the first (<34) and DL above the second (>70) tertiles, or when the ERI ratio resulted below the first tertile (<0.40). Nurses with strain scores not included in these categories were classified in a unique ‘intermediate job stress’ category. The work stress conditions over time were determined combining the results of the two surveys. Subjects who reported high work stress and low work stress in both surveys were categorized at “prolonged high stress” (PHS) and “stable low stress” (SLS), respectively. Finally, nurses in the intermediate work stress category in the first survey and at high work stress in the second survey, were considered in a category called “recent high stress” (RHS). The numbers of nurses in the SLS, RHS and PHS groups were 52, 9 and 20, respectively.

### 2.3. Exclusion Criteria for HRV Measurements

Based on available clinical records, some subjects were excluded for ECG monitoring due to being affected by conditions altering the autonomic nervous system (ANS), like established diagnoses of cardiovascular diseases, hypertension, neurological disorders, diabetes and other metabolic diseases or endocrinal disorders, pregnancy and lactation. Similarly, we excluded subjects taking drugs with effects on the ANS, like antihypertensive, antiarrhythmic, neuroleptic, antidepressant medications. 

**Figure 1:**
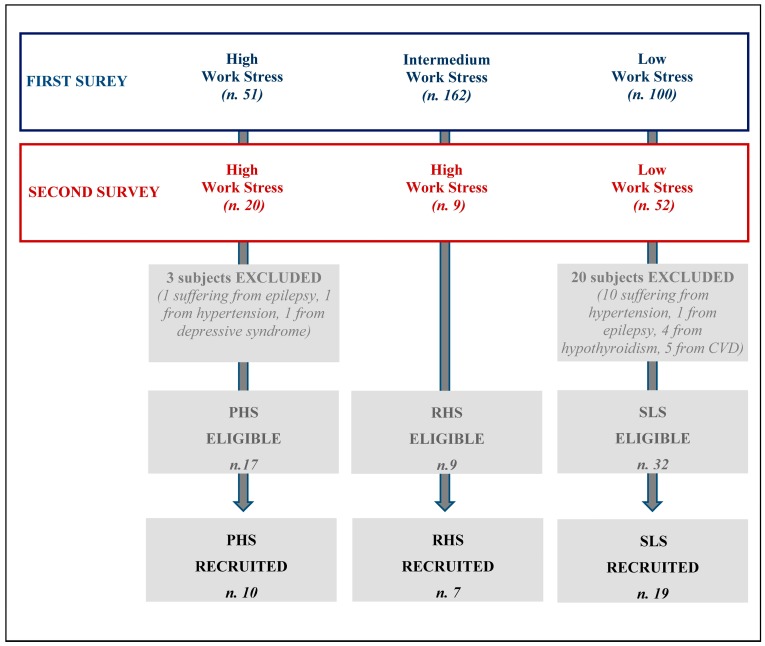
The flow-chart shows the selection of enrolled sample and the application of the exclusion criteria to each chronic work stress categories.

The flow-chart in Figure 1 shows the results of the application of these exclusion criteria to each work stress categories. Three subjects were excluded in the PHS group and 20 in the SLS group. Among the residual 17 PHS eligible nurses 10 gave their written consent to undergo ECG monitoring. Similarly, 19 out of the eligible 32 SLS and 7 of the 9 RHS nurses gave their written consent to take part the ECG monitoring. For this part of the study, the local hospital ethical committee was informed, but a formal approval at the time of recruitment (in 2010) was not required because of the observational and non-invasive nature of the study [34].

### 2.4. ECG Monitoring and HRV Analysis

All enrolled subjects underwent a twenty-four-hour ECG monitoring, repeated twice in a working day (WD) and a subsequent resting day (RD), within 1 month following the second survey. Working time covers the periods from 7 a.m. to 2 p.m. or from 2 p.m. to 9 p.m.

The ECG monitoring was carried out at least 72 h apart from a night shift to reduce the effect of sleep deprivation. Holter ECG recording was applied in the morning, between 7:00 and 8:00 a.m. and it was removed the following morning. All subjects were instructed to fill in a diary, indicating the starting and the ending times of each relevant activity (e.g., work, sleep, meals, rest, light physical activity) occurred during the ECG recordings. ECG Holter monitoring placement was performed by professional nurses, on workers in a semi-upright sitting position. Twelve channels were used in order to better detect rate and location of any abnormal repolarization [35]. ECG segments with anomalies, ectopic beats or artifacts were excluded from the HRV analysis, which was carried out by a single cardiologist according to the current standard guidelines [6]. The data acquired from a Mortara H Scribe 12 lead monitoring system were fed into a dedicated spectral analysis module (Mortara software for HRV analysis). This program uses a Fourier transformation that compute a power frequency spectrum from 0.01 to 0.50 Hz, separately for the low frequency (LF) in the range 0.04–0.15 Hz and the high frequency (HF) in the range 0.15–0.40 Hz, as absolute values of variance in milliseconds squared (in ms^2^). The LF and HF ratio was also computed.

For each subject, we registered 24-h ECG recordings for each day (WD and RD) [27]. For WD, registrations were separated in the working (WP), non-working (W-NWP) and night sleeping periods. For morning periods, we eliminated recordings before 9.00 a.m. to exclude the interferences of the cortisol peak. We also excluded periods with ECG artifacts, ectopic beats or transitory arrhythmias. For each daytime recording, we excluded periods of naps, strong physical activity and emotional disturbances (based on subjects’ reports in diaries). All these restrictions induced us to analyze 2-h continuous ECG registrations, as the minimum time periods common for all subjects and all periods of WDs and RDs. When longer periods remained available, the selection of the 2-h recordings was done identifying continuous periods with the lower heart rate, based on the consideration to further exclude excessive physical and emotional demands. Finally, the selection of the 2-h periods during the night sleeping time was not based on objective evaluations of sleep phases, which require the adoption of complex instrumentations, difficult to realize in real field studies.

### 2.5. Statistical Analysis

We summarized major demographic, clinical and psychological characteristics in the three work stress exposure categories (SLS, RHS, PHS) using either mean and standard deviation, or frequencies, for quantitative and qualitative variables, respectively. Differences across groups were tested through the ANOVA for quantitative variables and the chi-square test for qualitative variables. To reduce the influence of extreme values, HRV parameters were log-transformed (natural logarithm), and we report the geometric mean. As in previous analyses [27], we included age and current cigarette smoking as covariates in our models, while the high prevalence of women (83%) did not allow us considering the potential effect of gender on HRV frequency domains.

We estimated the age- and smoking status-adjusted geometric means for the HRV frequency domain parameters across the SLS, RHS and PHS groups from ANCOVA linear regression models, for registrations in the working day (working, non-working and night periods) and in the subsequent resting day (morning and night). We tested the differences across groups using a 2-df Wald chi-square test, while pairwise comparisons between groups were tested adjusting for multiplicity with the Tukey-Kramer method. Finally, we estimated within each exposure group, changes in HF and LF parameters using repeated-measures regression models, adjusted for age and smoking status, across a conceptual recovery time scale ranked from working periods of WDs, to non-working periods of a WDs and then to subsequent mornings of RDs. Separate beta-coefficients over time were estimated and the null hypothesis of no change (beta = 0) was tested using a Wald chi-square test. A two-sided α equal to 0.05 was the level of significance for all statistical tests. Statistical analyses were performed with SAS Version. 9.4 (SAS Institute, Inc., Cary, NC, USA), while the figure was drawn using R (3.2.5 version, R Foundation for Statistical Computing, Vienna, Austria).

## 3. Results

The final sample size consisted in 36 healthy nurses (*n* = 26, 72.2%) and nurse assistants (*n* = 10, 27.8%) who gave their written consent to participate, of which 19, 7 and 10 in the SLS, RHS and PHS groups, respectively (Figure 1).

Eligible and recruited nurses did not show statistically significant differences in major demographic and clinical characteristics (data not shown). Eligible and enrolled nurses were characterized by a high proportion of women (above 80%), to be mostly professional nurses (more than 70%) and with a mean age around 39 years and an average job tenure of about 11 years. As reported in Table 1, the three work stress groups did not differ for the proportion of females, professional nurses, level of education achieved, and for a positive family history of diabetes, ischemic heart disease and hypertension, as well as for the mean values of length of employment and BMI. A significant difference was found only for proportion of current smokers (*p*-value = 0.03), with a higher prevalence in the PHS group. Even if the mean age was not statistically different among the groups, the SLS groups was younger by three and almost four years in comparison to the RHS and PHS, respectively.

Table 2 shows the results of spectral analysis of HRV in different periods of the working and the resting days, for the work stress exposure groups. We observed a statistically significant difference for the HF power in the three groups during the working period of the working day (WD-WP), with the lowest HF values in the PHS (geometric mean: 76.3) and the highest in the SLS group (139.1); the pairwise comparison between these two groups showed a difference borderline statistically significant (*p* = 0.08). In the remaining analyzed periods of the WD (i.e., NWP and sleeping during night), as well as in the two periods of the RD, there were no statistically significant differences in HF, although the lowest values were consistently observed among the PHS category.

Statistically significant differences were observed among work stress groups for LF values in the working day, in WP (*p*-value = 0.02) and in NWP (*p*-value = 0.02), as well as in the subsequent resting morning (*p*-value 0.04). In particular, the geometric mean values were constantly halved in the PHS as compared to the SLS groups (for all pairwise comparison *p*-values < 0.05). Conversely, sleeping LF did not show alterations across stress groups, neither during the working nor in the resting days. Finally, we did not observe any difference in the LF/HF ratio across groups. This finding is consistent with the observed consensual changes of LF and HF values. 

**Figure 2:**
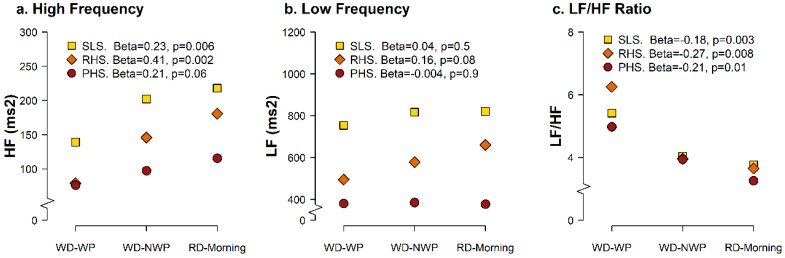
Age- and smoking-adjusted geometric mean for high frequency (HF) (panel **a**), low frequency (LF) (panel **b**) and LF/HF ratio (panel **c**) in consecutive periods of the working and of the resting days, and their linear trend over time (beta-coefficient) within each work stress group.

Figure 2 shows the age- and smoking-adjusted geometric means for HF (panel a), LF (panel b) and their ratio (panel c) in consecutive periods of the working day and of the subsequent resting morning, and their linear trend over time within each work stress group. HF values increase over time in both the SLS and the RHS groups (Figure 2, panel a), with beta values of 0.23 and 0.41, both statistically significant. PHS nurses showed borderline significant increase in the HF parameter, with a rate of recovery (beta = 0.21, *p* = 0.06) almost half of to one observed in the RHS. Such trends indicate that HF reduces during the working time in stressed nurses, but while the RHS group shows a clear tendency to increase back HF variability starting from the non-working period and continuing in the following rest day morning, this trend is attenuated for nurses in the PHS group, indicating that recovery is reduced.

Conversely, the changes over time of LF were flat in the SLS group (beta = 0.04, with stable high values) and in the PHS group (beta = −0.004, with permanent low values; Figure 2, panel b). In the RHS group, the estimated beta was 0.16, indicating some increase in LF in the non-working period and resting morning as compared to the working period, although it was borderline statistically significant (*p* = 0.08), but the small sample size should be take into account (*n* = 7). In comparison with the trends registered for HF, LF shows an absent recovery in the PHS group and a similar tendency to recover for the RHS subjects. Finally, the LF/HF ratio decreased over time in all the three stress categories (Figure 2, panel c), because of the steady and the increased trends in LF and HF, respectively. 

## 4. Discussion

With the purpose of assessing the effects of chronic work stress on cardiac autonomic control, we investigated twice, in a one-year interval, a sample of 36 healthy adult nurses, predominantly women. The spectral analysis of HRV of two-hour ECG recordings showed in the working periods a statistically significant difference of HF among the work stress groups, with lower mean values in the PHS and RHS groups, when compared to the SLS. These differences decrease in non-working and resting periods. Based on the same two-hour ECG recordings, we also reported lower mean values of LF in stressed nurses during working time. This reduced HRV in the low frequency power tended to recover in nurses under recent stress conditions over a time period covering the non-working period of the same working day and the following resting period. Instead, in nurses under prolonged working stress conditions, the recovery did not take place, and the decreased LF power stabilized even in non-working and resting periods. This may explain the detrimental effects on the cardiovascular system, previously repeatedly documented [1,2]. Finally, a decrease over time of LF/HF ratio was observed in all the three groups, driven only by the increase of HF power during the resting periods.

A plausible pathophysiological mechanism of this relationship advocates for disturbances of the autonomic nervous system based on a disequilibrium of the sympathetic and the vagal branch [4,5,6].

Many authors have found an inverse association between high levels of work stress and HF values [20,21,23,26,29,32]. Chandola et al. found low job control was associated with lower HF means [20,21]. Similarly, Collins et al. reported that low job control was associated with lower HF in a sample of 36 healthy men, healthcare employees [23]. Hintsanen et al. in a sample of 863 healthy Finnish workers found that high perceived work stress, assessed by the ERI questionnaire, was associated with a lower HF in woman, but not in men [29]. In a Dutch study on 70 healthy workers, Hanson et al. found significant decrements of HF in the subjects with a high ERI ratio [32]. Hernandez-Gaytan et al. in a sample of 54 healthy men and women, resident physicians in a Mexico City hospital, showed a lower HF in passive subjects, based on the JCQ questionnaire [26]. 

The association between work stress and LF values at present is more controversial. Chandola et al. [20,21] found lower LF means in high job strain healthy workers [20,21]. Similar results were obtained by Hernandez-Guytan et al. [26] in high work stress conditions. Conversely, in a sample of 135 healthy men and women from different job titles, van Amelsvoort et al. [31] founded higher LF values among JCQ passive and active subjects, but he presented his results in normalized unit of LF. It has been recently reported that such normalized values can lead to data distortions, introducing possible biases [36]. Kang et al., in a sample of 169 Korean subjects, evidenced lower HF and LF values in high job strain healthy male blue collars, but not statistically significant [25]. Kageyama et al. found a significant reduction of the HF component only in stressed subjects with also sleep disturbances [30].

It is known that the HRV HF component characterizes the vagal control on the heart [6]. Malliani et al. originally gave evidences in support of the theory that the LF power can be considered as an index of sympathetic cardiac control [37]. More recently, other authors have suggested that LF power characterizes both sympathetic and parasympathetic influences on the heart functions [6,7]. Finally, a recent review, with a reanalysis of the results reported by previous reports, suggested that the entire HRV power spectrum, including the LF component, is mainly determined by the parasympathetic system [36]. In this respect, low HRV frequency domain parameters can be considered as a possible marker of the decreased parasympathetic tone, connecting stress and the negative consequences on the heart. In light of these recent interpretations, our findings add further support to the hypothesis that disturbances of the autonomic nervous system, and a reduction in the vagal control of the heart rate in particular, can play an important role in the mechanisms linking work stress and cardiovascular effects. For this purpose, it is worth mentioning that the same alterations of the autonomic control have been reported by Collins et al. in stressed and exhausted subjects [38].

The first strength of our study is to be one-year longitudinal, and therefore able to characterize prolonged perceived work stress conditions, and in effect the main alterations of the FD HRV could be detected exactly in this exposure group which connotes chronic stress. To our knowledge, only one study adopted a one-year longitudinal study, but the authors did not report HRV results in the frequency domains [28]. Another strength is that we monitored HRV during separate periods a working and resting day, allowing us to assess recovery time of HRV alterations. A further strength of the present study is the careful application of exclusion criteria, which helped in avoiding the effects on HRV from known diseases and drug treatments. Finally, in our view the adoption of two separate scores for detecting the perceived work stress, i.e., ERI and JCQ, allowed to reduce misclassifications in classifying exposed and unexposed subjects. 

The major shortcoming of our study is the small sample size, in particular in the recently strain group. The reduced statistical power may then be responsible of poor capacity to detect some differences and trends that we found at a borderline statistical significance. As such, our study results need replication on larger samples. Finally, our frequency domain HRV analysis was done on continuous two-hour ECG recordings, in compliance with current guidelines [6]. In Section 2, we reported how the analyzed periods were obtained from longer registrations when available. Other studies used either longer or shorter time periods. In real field studies, the effects of using selected time periods when longer ECG recordings are available needs to be further investigated. 

## 5. Conclusions

In conclusion, based on a one-year longitudinal study, our results evidence that only prolonged perceived high work stress (i.e., lasting at least one year) is associated with consistent reductions of both HF and LF powers. Moreover, under persistent stressful working conditions, recovery to higher HF and LF values during not-working and resting periods is reduced or even absent. These alterations of the two major HRV power components under prolonged work stress conditions are in accordance with a recent review of autonomic cardiovascular regulation studies indicating that HF and LF are both determined by the parasympathetic system.

## Figures and Tables

**Table 1:** Demographic and clinical characteristics in the investigated three strain categories: stable low strain (SLS); recently high strain (RHS) and prolonged high strain (PHS).

Variable	Statistic	SLS (*n* = 19)	RHS (*n* = 7)	PHS (*n* = 10)	*p*-Value
Age (years)	*Mean* (*SE*)	37.3 (1.9)	40.3 (2.8)	41.0 (4.1)	0.58 **^§^**
Female	*n* (%)	15 (79.0)	6 (85.7)	9 (90.0)	0.74 ^
Professional Nurses	*n* (%)	16 (84.2)	4 (57.1)	6 (60.0)	0.23 ^
Bachelor Degree	*n* (%)	7 (36.8)	2 (28.6)	2 (20.0)	0.64 ^
Length of Employment (years)	*Mean* (*SE*)	10.2 (2.1)	7.1 (1.3)	13.7 (4.2)	0.40 **^§^**
Positive family history of Diabetes	*n* (%)	9 (47.4)	3 (42.9)	4 (40.0)	0.93 ^
Positive family history of Hypertension	*n* (%)	14 (73.7)	2 (28.6)	8 (80.0)	0.06 ^
Positive family history of CHD	*n* (%)	10 (52.6)	3 (42.9)	4 (40.0)	0.78 ^
Body Mass Index (BMI)	*Mean* (*SE*)	24.4 (0.8)	25.4 (1.8)	22.1 (1.1)	0.18 **^§^**
Current Cigarette Smokers	*n* (%)	5 (26.3)	0 (0.0)	6 (60.0)	0.03 ^

Legend: ^§^ = F-test from ANOVA and ^ Chi-square test. SE = Standard Error.

**Table 2:** Age- and smoking-adjusted geometric mean (95% CI) for Frequency Domain HRV parameters in different periods of the working and of the resting days, by work stress groups.

FD HRV	Day	Period	Work Stress Exposure Groups			Wald Chi-Square ^	*p*-Value ^	Pairwise Comparison *p*-Values *		
			SLS (*n* = 19)	RHS (*n* = 7)	PHS (*n* = 10)			SLS vs. RHS	SLS vs. PHS	RHS vs. PHS
**HF (ms^2^)**	**WD**	**WP**	139.1 (100.7; 192.1)	79.1 (46.5; 134.6)	76.3 (48.9; 119.1)	5.98	**0.05**	0.2	0.08	1.0
		**NWP**	202.1 (135.7; 301.1)	146.0 (75.7; 281.6)	97.6 (56.3; 169.0)	4.46	0.11	0.7	0.09	0.6
		**Night**	635.0 (366.3; 1100.6)	455.0 (183.8; 1126.0)	368.0 (172.4; 785.4)	1.38	0.5	0.8	0.5	0.9
	**RD**	**Morning**	217.9 (142.7; 332.9)	180.9 (90.0; 363.5)	115.6 (64.5; 207.3)	2.97	0.2	0.9	0.2	0.6
		**Night**	721.7 (457.4; 1138.6)	508.4 (239.9; 1077.7)	416.6 (222.2; 781.0)	2.07	0.4	0.7	0.3	0.9
**LF (ms^2^)**	**WD**	**WP**	753.3 (560.0; 1013.4)	494.7 (303.5; 806.4)	380.2 (252.6; 572.1)	7.49	**0.02**	0.3	**0.02**	0.7
		**NWP**	816.7 (596.9; 1117.5)	577.8 (344.7; 968.6)	384.5 (249.6; 592.4)	7.73	**0.02**	0.5	**0.02**	0.5
		**Night**	1020.2 (646.8; 1609.1)	590.5 (278.7; 1251.0)	650.8 (347.3; 1219.6)	2.13	0.3	0.4	0.5	1.0
	**RD**	**Morning**	820.2 (575.9; 1168.2)	660.9 (369.1; 1183.6)	377.1 (231.6; 614.1)	6.42	**0.04**	0.8	**0.03**	0.3
		**Night**	1002.8 (672.5; 1495.3)	581.0 (300.8; 1122.1)	574.1 (331.0; 995.7)	3.47	0.2	0.3	0.2	1.0
**LF/HF**	**WD**	**WP**	5.4 (4.2; 7.0)	6.3 (4.1; 9.6)	5.0 (3.5; 7.1)	0.63	0.7	0.8	0.9	0.7
		**NWP**	4.0 (3.1; 5.2)	4.0 (2.6; 6.1)	3.9 (2.8; 5.6)	0.01	1.0	1.0	1.0	1.0
		**Night**	1.6 (1.3; 2.0)	1.3 (0.9; 1.9)	1.8 (1.3; 2.5)	1.39	0.5	0.6	0.9	0.5
	**RD**	**Morning**	3.8 (2.8; 5.1)	3.7 (2.2; 6.1)	3.3 (2.1; 5.0)	0.28	0.9	1.0	0.9	0.9
		**Night**	1.4 (1.1; 1.8)	1.1 (0.8; 1.7)	1.4 (1.0; 1.9)	0.77	0.7	0.7	1.0	0.7

Abbreviations: WD, Working Day; RD, Resting Day; WP, Working period in the Working Day; NWP, Non-Working period in the Working Day; WD-N, Night sleeping in the Working Day; RD-Morning, morning in the Resting Day; RD-Night, Sleeping in Night in the Resting Day. ^: Wald Chi-square test testing the null hypothesis of no change in HRV parameter among stress groups (2-df test). *: *p*-value comparisons: adjustment for multiple comparisons according to Tukey-Kramer method. Data in **bold**: *p* < 0.05.
